# Clinicopathological significance and prognostic role of LAG3 + tumor-infiltrating lymphocytes in colorectal cancer; relationship with sidedness

**DOI:** 10.1186/s12935-023-02864-3

**Published:** 2023-02-10

**Authors:** Shirin Tavana, Zahra Mokhtari, Mohammad Hossein Sanei, Zahra Heidari, Amir-Reza Dehghanian, Zahra Faghih, Marzieh Rezaei

**Affiliations:** 1 Department of Immunology, School of Medicine, Isfahan University of Medical Sciences, Isfahan, 8174673461 Iran; 2 Department of Pathology, School of Medicine, Isfahan University of Medical Sciences, Isfahan, Iran; 3 Department of Biostatistics and Epidemiology, School of Health, Isfahan University of Medical Sciences, Isfahan, Iran; 4 Department of Pathology, School of Medicine, Shiraz University of Medical Sciences, Shiraz, Iran; 5 School of Medicine, Shiraz Institute for Cancer Research, Shiraz University of Medical Sciences, Shiraz, Iran

**Keywords:** Colorectal cancer, Immunohistochemistry, Prognostic marker, Tumor microenvironment, LAG3, CD45RO, CD3, CD8

## Abstract

**Background:**

It is well-documented that the interplay between tumor-infiltrating lymphocytes (TILs) and tumor cells is a major determining factor in cancer progression. CD45RO seems to be a reliable indicator for predicting prognosis and disease outcome, along with CD3 and CD8 markers. LAG-3 is another important marker that overexpresses on TILs in a variety of cancers and is associated with disease prognosis; however, its prognostic impact is controversial. Hence, in the present study, we aimed to investigate the presence of CD45RO + , LAG3 + , CD3 + , and CD8 + lymphocytes in CRC tumor tissues and their association with clinicopathological parameters of the disease as well as patients' survival, according to primary tumor locations.

**Methods:**

Expression of CD45RO, LAG3, CD3, and CD8 was immunohistochemically assessed in tissue sections of 136 patients with CRC. The percentages of TILs expressing these markers were then separately determined in both invasive margin (IM) and center of tumor (CT). Their associations with clinicopathological factors and patients’ survival were analyzed in the entire cohort and the subgroups of patients with right- and left- rectum tumors.

**Results:**

Based on our observation, CD45RO + and CD3 + cells were the most frequent infiltrated lymphocytes in both CT and IM regions of colon tumor tissue. Whilst, LAG3 + lymphocytes were the least frequent subset in both areas. Statistical analysis indicated that the frequency of CD45RO + TILs was positively associated with advanced TNM stages (III/IV), in the entire cohort and right-sided tumors (P < 0.05). LAG3 + TILs in IM were also increased in tumor tissues with higher T-stages in the entire cohort (P = 0.027). In univariate analysis, high score of CD45RO + TILs in IM was associated with better overall survival in the entire cohort. High score of CD8 + and CD45RO + lymphocytes in IM were also associated with improved survival in patients with right-sided tumors.

**Conclusions:**

Our findings generally suggest that the clinicopathological and prognostic significance of immune system-related markers such as CD45RO and LAG3 depends on the primary tumor sides. Our results collectively demonstrated that infiltration of CD45RO + lymphocytes in IM could be an independent prognostic factor in a site-dependent manner.

**Supplementary Information:**

The online version contains supplementary material available at 10.1186/s12935-023-02864-3.

## Introduction

Colorectal cancer (CRC), the second most common causes of cancer death in the world [1], can be identified by the location of primary tumor in the large intestine: right and left colon, and rectum [2]. Based on the location, CRC shows differences in chromosomal and molecular characteristics, microbiomes, incidence, pathogenesis, and outcome [3]. It has been shown that tumors on the left and right sides have also different immune landscapes. In this regard, the level of immune infiltration is higher in right-sided CRC than in left-sided tumors; thus, the prognosis may be different based on primary tumor locations [4].

CRC prognosis is mainly determined by histopathological grading, according to the American Joint Committee on Cancer (AJCC) TNM system [5]. Nevertheless, significant differences in clinical outcomes are commonly observed among patients with the same stage, showing the weaknesses of TNM classification system [6]. One of the failure reasons for this system seems to be the lack of attention to the impact of the tumor microenvironment (TME), especially the immune system elements, on tumor progression [7]. TME is generally defined as the environment surrounding the tumor, including the extracellular matrix, blood vessels, endothelial cells, tumor cells, and immune cells [8]. Therefore, the identification of immune-related markers in TME might help to predict the prognosis and clinical outcome of the disease.

Tumor-infiltrating lymphocytes (TILs) are the main immune cells of TME, and their presence is generally considered an indication of host immune reaction to tumor cells to prevent tumor invasion [9]. TILs compose of various subpopulations of CD3 + T cells [10] i.e. CD4 + and CD8 + T lymphocytes, FOXP3 + regulatory T cells, and CD45RO + memory T cells [11]. There are many reports that suggest that CD3 + , CD8 + , and CD45RO + TILs are correlated with favorable clinical outcomes in several cancers [12–14] due to their critical roles in tumor immunity [15]. However, the inhibitory microenvironment suppressed their effector function in favor of tumor progression.

CD45RO + memory T lymphocytes, as one of the essential components of TILs, are responsible for more reinforced responses to secondary antigens exposure [16]. Consistently, there are several reports that CD45RO + T cell infiltration had a positive prognostic effect on many types of tumors such as gastric, cervical, and hepatocellular carcinoma, though the accurate clinical significance of these T cells was not clear [12, 17].

Immune checkpoints (ICPs) are membrane proteins that regulate immune cells by either activating or inhibiting their immune functions and have the potential to act as prognostic biomarkers [18]. Inhibitory ICPs like T-cell immunoglobulin mucin-domain containing-3 (TIM-3), programmed cell death protein-1 (PD-1), and lymphocyte activation gene 3 (LAG3), are all present on T-cells and bind to the ligands that are commonly produced by tumor cells (18). Of them, LAG3 or CD223 is expressed on T cells, especially on activated CD8 + and CD4 + T cells, B cells, and DCs [19]. Interaction of LAG3 with its ligands i.e., MHC class II molecules, galectin-3, liver sinusoidal endothelial cell lectin (LSECtin), and fibrinogen-like protein 1 (FGL1) [20], mediates various signalings leading to impairment of TILs functions, including inhibition of Th1 proliferation and reduced production of interleukin-2 (IL-2), Interferon-γ, and tumor necrosis factor (TNF) in T cells, resulting in tumor escape [21]. LAG3 expression is frequently associated with exhausted T cells, and accordingly considered an exhaustion marker for CD4 + and CD8 + T cells in response to repetitive antigen stimulation in cancer [22–25]. Overexpression of LAG3 is found in many types of human tumors such as CRC, breast cancer, liver carcinoma, follicular lymphoma, and squamous cell carcinoma of the head and neck, and in most cases, is significantly correlated with the development of an invasive tumor and worse outcome [26–28].

However, there are limited studies, that showed the association of tumor-infiltrating LAG3 + T cells with CRC prognosis, their results were controversial. In addition, the majority of these studies investigate the prognostic impact of the total population of TILs, and fewer studies have focused on the prognostic significance of specific subpopulations. Accordingly, we aimed to evaluate the presence of CD45RO + and LAG3 + TILs in the CT and IM and their impacts on outcomes in CRC with particular focus on tumor location (right and left colon tumors).

## Materials and methods

### Patients and specimens

One hundred and thirty-six patients with CRC who had complete data were enrolled in the study. The study population was retrospectively selected from patients who underwent surgical resection of their primary tumors between April 2013 to December 2016 in Al-Zahra hospital (Isfahan University of Medical Sciences, Isfahan, Iran). Exclusion criteria were receiving preoperative chemotherapy, having a history of other cancers or autoimmune diseases, and insufficient and inappropriate tissue. Information on survival status (dead/alive), date and type of recurrence (if present), metastatic organ, and for patients who died, date and cause of death were collected from their records and telephone contact. Pathological data, including TNM-stage, histological grade, lymph node involvement, and lymphovascular or perineural invasion, were collected from patient files. An expert pathologist reviewed the H&E-stained tumor sections to confirm the patient's pathological data and select suitable blocks, preferably having both CT and IM. Formalin-fixed and paraffin-embedded (FFPE) tissue blocks were then selected and prepared for immunohistochemical staining.

### Immunohistochemistry

Tissue sections with 3-μm thickness were prepared and fixed on slides coated with poly-L-lysine. The sections were dewaxed at 44 °C overnight, deparaffinized in xylene, and rehydrated in a graded series of ethanol (100% and 96%). Antigen retrieval was then performed by boiling the tissue sections in a pressure cooker for 20 min in Tris–EDTA buffer (pH: 9.0). Endogenous peroxidase activity was blocked by incubation in 10% hydrogen peroxide (Master Diagnostica, Spain) for 10 min, and subsequently, the sections were blocked with 10% goat serum (Cyto Matin Gene, Iran) at room temperature for 30 min. Primary anti-human antibodies against LAG3 (1:1000, clone: EPR20261; Abcam, USA), CD45RO (1:320, clone UCHL1; Biolegend, USA), CD3 (1/2.5, clone: EP41; Master Diagnostica), CD8 (1:150, clone: C8/144B, Biolegend), were separately added and followed by overnight incubation at 4 °C. The slides were then washed and incubated with a horseradish peroxidase-conjugated secondary antibody for 40 min. The chromogenic reaction was visualized by incubation with diaminobenzidine (DAB) (Master Diagnostica) for 3–5 min. Sections were counterstained with hematoxylin, dehydrated by increasing alcohol solutions, cleared in xylene, and mounted with a permanent mounting medium. Human tonsil tissue with abundant lymphocytes, was used as a positive control to titrate the proper concentration of primary antibodies.

### Evaluation of immunochemistry staining

Two expert pathologists blindly reviewed the sections. The slides related to each patient were first examined with a magnification of 100 × , and normal area, invasive margin (IM), and tumor center (TC) were identified, and an area representing the average expression of the marker was selected. The selected area was then examined in a high-power field (× 400), and the percentage of CD45RO, LAG3, CD3, and CD8 expressing lymphocytes were determined. The percentage indicated the ratio of the positive cells to total lymphocytes. In addition to the percentages, the intensity of LAG3 expression in each region was also reported as negative, low, moderate, and high. LAG3 expression was also scored by multiplying the number of LAG-3 + TILs by the intensity using R software (version 4.0.3). Accordingly, the patients were classified into high score (LAG3 ^hi^) and low score (LAG3 ^Low^) groups. The scores for CD3, CD8, and CD45RO were determined based on their median expression and patients were classified into high and low score groups.

### Statistical analysis

Statistical analyses were performed using SPSS software version 24 (IBM SPSS, USA). The normality of CD45RO and LAG3 expression in different groups was checked. The significance of differences in CD45RO and LAG3 expression between several clinicopathological variables was assessed by t-test, non-parametric Mann–Whitney test, Chi-square test or Fisher’s exact test as appropriate. The Chi-square test was performed in the entire cohort and based on primary tumor location (right-sided and left-sided colon tumors). Survival times were defined as overall survival (OS): the time between surgery and cancer-related death, and disease-free survival (DFS), between the surgery and any type of relapse or last follow-up. For univariate analyses, the Cox regression method was used to determine predictors of DFS and OS in patients with CRC. Those variables in the univariate analysis that had a P < 0.1 were included in the multivariate Cox’s regression model to investigate the independent predictive factors. P < 0.05 were considered statistically significant.

## Results

### Patients’ characteristics

The final study population consisted of 136 patients with CRC with the mean age (± SD) of 62.35 (± 14.10) years (19–92 years) at the time of diagnosis. According to the pathology reports, 109 (80.1%) patients had colonic tumors; of them, 56 (41.2%) tumors were located on the right side, and 49 (36%) ones were on the left side. Based on pathologic stages, most patients were in stage II (33.8%) and stage III (29.4%). Distant metastasis was seen in 12 patients (8.8%) (M1), and 46 patients (33.8%) had lymph node involvement at the time of surgery. Regarding tumor histological grade, most cases (76/136, 55.9%) had well-differentiated tumors.

The mean ± SD follow-up period was 54.08 ± 27.6 months. During follow-up period, 57 (41.9%) patients died, and 79 (58.1%) cases were alive up to October 2021. During this time, 30 patients (22.1%) experienced recurrence. The summarized clinical and pathological information of the patients are listed in detail in Table 1.

**Table 1:** Clinicopathological characteristics of patients with colorectal cancer

Parameters	No. of cases (%)	Mean ± SD	Parameters	No. of cases (%)
Total	136		TNM stage	
Sex			I/II	84 (61.8)
Male	83 (61.0)		III/IV	52 (38.2)
Female	53 (39.0)		Lymphovascular invasion (LVI)	
Age		62.35 ± 14.102	Absent	79 (58.1)
< 63	65 (47.8)		Present	57 (41.9)
≥ 63	71 (52.2)		Perineural invasion	
Tumor side			Absent	111 (81.6)
Right	56 (41.2)		Present	25 (18.4)
Left	76 (55.9)		Metastasis	
Unreported	4 (2.9)		Absent	89 (65.4)
Tumor size		5.56 ± 2.47	Present	34 (25.0)
< 5	56 (41.2)		Unreported	13 (9.6)
≥ 5	78 (57.4)		Recurrence	
Unreported	2 (1.5)		Absent	97 (71.3)
Differentiation grade			Present	29 (21.3)
Low grade	76 (55.9)		Unreported	10 (7.4)
Moderate to high grade	60 (44.1)		Tumor budding	
T stage			Low	89 (65.4)
T1/T2	47 (34.6)		High	47 (34.6)
T3/T4	89 (65.4)		Tertiary lymphoid structure (TLS)	
Lymph node involvement			Absent	104 (76.5)
Absent	90 (66.2)		Present	32 (23.5)
Present	46 (33.8)		Survival	
M stage			Alive	79 (58.1)
M0	124 (91.2)		Dead	57 (41.9)
M1	12 (8.8)			

### Distribution of CD45RO + , LAG3 + , CD3 + , and CD8 + TILs in patients with CRC

**Fig. 1:**
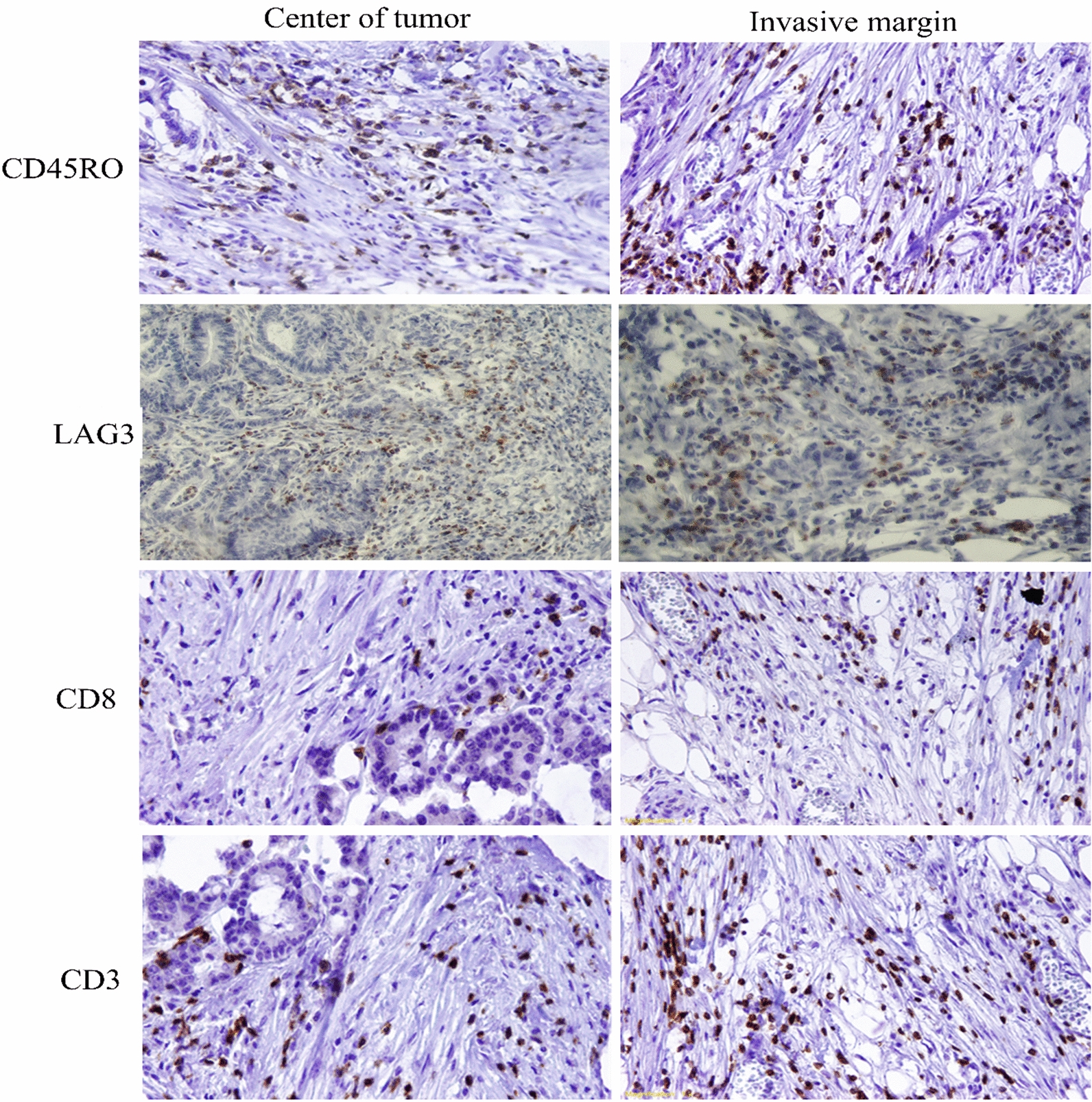
Immunohistochemical staining pattern of CD3, CD8, LAG3, and CD45RO in colorectal cancer tissues. Center of tumor (CT) and Invasive margin (IM) regions. To create the best conditions for antigen retrieval and optimal dilution of primary antibodies, staining was first performed on human tonsil tissue with different dilutions of anti-CD3, anti-CD8, anti-CD45RO, and anti-LAG3 antibodies. The same method was performed with the same dilutions on CRC tissue and finally, 1/2.5 dilutions for CD3 marker, 1/150 for CD8 marker, 1/320 for CD45RO marker, and 1/1000 for LAG3 marker were determined as optimal dilutions. The figure shows the staining patterns of these markers with selected dilutions in different regions of CRC tissue

Tissue samples were immunohistochemically stained for CD45RO, LAG3, CD3, and CD8 (Fig. 1). The mean frequencies of CD45RO + , LAG3 + , CD3 + , and CD8 + TILs were assessed in both CT and IM as well as normal-like adjacent tissues (if had indication). Our analysis indicated that the mean frequency of all markers in both CT and IM was significantly higher than in normal-like tissue. The mean frequency of CD45RO + lymphocytes in the IM area was significantly higher than CT (P < 0.001). On the other hand, the mean frequency of LAG3 + lymphocytes in CT was significantly higher than normal tissue. (Table 2).

**Table 2:** Mean frequency of CD45RO + , LAG3 + , CD3 + , and CD8 + lymphocytes in patients with colorectal cancer

		Number	Mean ± SD	P-value
Pair 1	CD45RO.CT	136	45.7721 ± 13.99575	< 0.001***
	CD45RO.IM	136	51.8750 ± 12.54529	
Pair 2	CD45RO.CT	47	46.8085 ± 13.32803	< 0.001***
	CD45RO.Normal	47	36.4894 ± 12.41996	
Pair 3	CD45RO.IM	47	52.2340 ± 11.64738	< 0.001***
	CD45RO.Normal	47	36.4894 ± 12.41996	
Pair 4	LAG3.CT	66	5.4632 ± 11.13680	0.571
	LAG3.IM	66	5.4485 ± 9.13019	
Pair 5	LAG3.CT	16	6.4237 ± 12.10187	0.026*
	LAG3.Normal	16	1.1695 ± 2.19837	
Pair 6	LAG3.IM	15	6.1356 ± 10.79025	0.058
	LAG3.Normal	15	1.1695 ± 2.19837	
Pair 7	CD3.CT	118	39.8729 ± 12.23806	< 0.001***
	CD3.IM	118	46.1017 ± 12.10946	
Pair 8	CD3.CT	51	40.8824 ± 12.59785	< 0.001***
	CD3.Normal	51	27.3529 ± 10.74025	
Pair 9	CD3.IM	51	46.8627 ± 11.17859	< 0.001***
	CD3.Normal	51	27.3529 ± 10.74025	
Pair 10	CD8.CT	119	19.1597 ± 9.44025	< 0.001***
	CD8.IM	119	24.0756 ± 9.52175	
Pair 11	CD8.CT	49	19.0816 ± 9.27999	0.002**
	CD8.Normal	49	15.0000 ± 7.56913	
Pair 12	CD8.IM	47	25.2128 ± 9.02638	< 0.001***
	CD8.Normal	47	14.7872 ± 7.65829	

*CT* Central of tumor, *IM* Invasive margin

^*^p < 0.05,**p < 0.01, ***p < 0.001

### Mean frequency of CD45RO + and LAG3 + lymphocytes in patients with different clinicopathological features

The mean frequency of CD45RO + , LAG3 + , CD3 + , and CD8 + lymphocytes was then evaluated in patients with different clinicopathological features. However, no significant relationships were observed between the mean frequency of CD3 + and CD8 + cells and clinicopathological features, as shown in Table 3, the mean frequency of CD45RO + lymphocytes in CT, was higher in patients with lymph node metastasis (P = 0.016) and those with advanced TNM-stages (III/IV) (P = 0.037) comparing to node-negative and lower-staged patients (I/II), respectively. In addition, the mean frequency of CD45RO + TILs in IM of females was higher than males (P = 0.041). The mean frequency of LAG3 + TILs in CT was observed to be higher in patients with lower T-stages (T1/T2) (P = 0.025) and no metastasis (P = 0.055). The mean frequency of these lymphocytes in IM was higher in patients with larger tumor size (≥ 5 cm) than those with lower tumor size (< 5 cm) (P = 0.024).

**Table 3:** Mean frequency of CD45RO + and LAG3 + lymphocytes in patients with different clinicopathological features

Parameters	CD45RO.CT		CD45RO.IM		LAG3.CT		LAG3.IM	
	Mean ± SD	P-value	Mean ± SD	P-value	Mean ± SD	P-value	Mean ± SD	P-value
Sex								
Male	45.00 ± 14.86	0.903ª	50.12 ± 12.90	0.041*	4.95 ± 10.06	0.696	5.27 ± 8.46	0.907
Female	46.98 ± 12.57		54.62 ± 11.55		6.26 ± 12.70		5.71 ± 10.17	
Age								
< 63	46.00 ± 13.11	0.857	52.00 ± 11.95	0.912	5.35 ± 11.22	0.531	4.47 ± 8.07	0.407
≥ 63	45.56 ± 14.84		51.76 ± 13.15		5.56 ± 11.13		6.33 ± 9.97	
Tumor side								
Right	45.00 ± 13.75	0.448	49.55 ± 12.90	0.263ª	5.32 ± 11.38	0.807	6.96 ± 12.28	0.322
Left	46.84 ± 13.75		53.42 ± 12.28		5.64 ± 11.20		4.39 ± 5.99	
Tumor size								
< 5	44.82 ± 13.31	0.555	53.30 ± 13.11	0.240	6.21 ± 12.91	0.809	3.44 ± 6.95	0.024*
≥ 5	46.28 ± 14.60		50.70 ± 12.16		5.06 ± 9.85		6.96 ± 10.29	
Differentiation grade								
Low grade	44.08 ± 12.16	0.113	52.17 ± 10.40	0.768	4.42 ± 9.84	0.369ª	5.38 ± 9.41	0.841
Moderate to high grade	47.91 ± 15.87		51.50 ± 14.91		6.78 ± 12.54		5.53 ± 8.83	
T stage								
T1/T2	44.46 ± 11.52	0.432	51.91 ± 9.97	0.979	6.42 ± 11.66	0.025*^,^ª	5.23 ± 6.58	0.603
T3/T4	46.46 ± 15.15		51.85 ± 13.76		4.95 ± 10.88		5.56 ± 10.26	
Lymph node involvement								
Absent	43.72 ± 13.50	0.016*	52.44 ± 12.92	0.461	6.15 ± 11.92	0.422	6.35 ± 10.63	0.335
Present	49.78 ± 14.22		50.76 ± 11.83		4.11 ± 9.40		3.67 ± 4.66	
M stage								
M0	45.52 ± 14.07	0.859ª	51.97 ± 12.78	0.556ª	5.45 ± 11.16	0.911ª	5.68 ± 9.44	0.278
M1	48.33 ± 13.54		50.83 ± 10.18		5.58 ± 11.36		3.00 ± 4.43	
TNM stage								
I/II	43.80 ± 13.85	0.037*	52.55 ± 13.27	0.421	6.38 ± 12.25	0.388	6.65 ± 10.92	0.200
III/IV	48.94 ± 13.77		50.77 ± 11.30		3.98 ± 8.95		3.5000 ± 4.50	
Lymphovascular invasion								
Absent	44.68 ± 12.20	0.287	51.58 ± 12.70	0.750	5.21 ± 11.01	0.301	5.99 ± 9.30	0.709
Present	47.28 ± 16.15		52.28 ± 12.43		5.80 ± 11.39		4.70 ± 8.91	
Perineural invasion								
Absent	44.59 ± 13.64	0.212ª	51.75 ± 12.73	0.818	5.63 ± 11.62	0.952	5.89 ± 9.84	0.667
Present	51.00 ± 14.65		52.40 ± 11.91		4.72 ± 8.80		3.48 ± 4.46	
Metastasis								
Absent	45.22 ± 13.35	0.595	51.85 ± 11.99	0.663	6.33 ± 12.30	0.055ª	6.28 ± 10.35	0.064
Present	46.76 ± 16.69		50.73 ± 14.41		3.03 ± 7.22		2.97 ± 4.21	
Recurrence								
Absent	45.10 ± 13.55	0.592	51.65 ± 12.18	0.928	6.49 ± 12.36	0.040*	6.38 ± 10.06	0.131
Present	46.72 ± 16.49		51.89 ± 15.20		1.86 ± 2.86		2.96 ± 4.01	
Survival								
Alive	44.87 ± 13.89	0.380	53.54 ± 12.61	0.068	6.28 ± 12.68	0.366	6.16 ± 10.85	0.158
Dead	47.01 ± 14.16		49.56 ± 12.18		4.33 ± 8.52		4.45 ± 5.92	

*CT* Central of tumor, *IM* Invasive margin

ªP-values were obtained from non-parametric Mann–Whitney test.*p < 0.05

### Associations of CD45RO + and LAG3 + TILs with clinicopathological features

We next scored CD45RO, LAG3, CD3 and, CD8 markers (as described in "Evaluation of immunochemistry staining" Section) and classified patients into high- and low-scores. Investigating the association of these scores with different clinicopathological features, revealed no significant relationship was observed between the score of CD3 and CD8 markers in CT and IM and clinicopathological features.

Association between the score of CD45RO and LAG3 in both CT and IM and clinicopathological characteristics (detailed in Additional file 1: Tables S1, S2). Our results also demonstrated that the score of CD45RO in CT was lower in patients without lymph node involvement (P = 0.013), and those with earlier TNM-stages (I/II) (P = 0.031) comparing to node-negative and lower-staged patients (I/II), respectively. 

In the case of LAG3, low score of LAG3 in CT was significantly associated with no metastasis (P = 0.023) and no recurrence (P = 0.015). While, the score of LAG3 in IM was observed to be higher in patients with higher T-stages (T3/T4) (P = 0.027), the absence of TLS in CRC tissues (P = 0.014), and larger tumor size (P = 0.052).

### Associations of CD45RO + and LAG3 + TILs with clinicopathological features based on primary tumor location

We then classified patients based on their primary tumor location into two groups: left/rectum- and right- sided tumors, and repeated the analyses in patients with high and low scores of LAG3 and CD45RO. Our results indicated that in right-sided tumors, low score of CD45RO in CT was associated with earlier TNM-stages (I/II) (P = 0.017) and the absence of lymph node involvement (P = 0.015).

In left-sided colon tumors, low score of LAG3 in CT was significantly associated with no metastasis (P = 0.039), no recurrence (P = 0.027), and low tumor budding (P = 0.05). The score of LAG3 in IM was lower in patients with higher T-stage (T3/T4) (P = 0.015) in these tumors. Detailed associations between the score of CD45RO and LAG3 in CT and IM and clinicopathological characteristics based on primary tumor location are demonstrated in Additional file 1: Tables S1, S2.

### Prognostic value of LAG3 + , CD45RO + , CD3 + , and CD8 + TILs

**Fig. 2:**
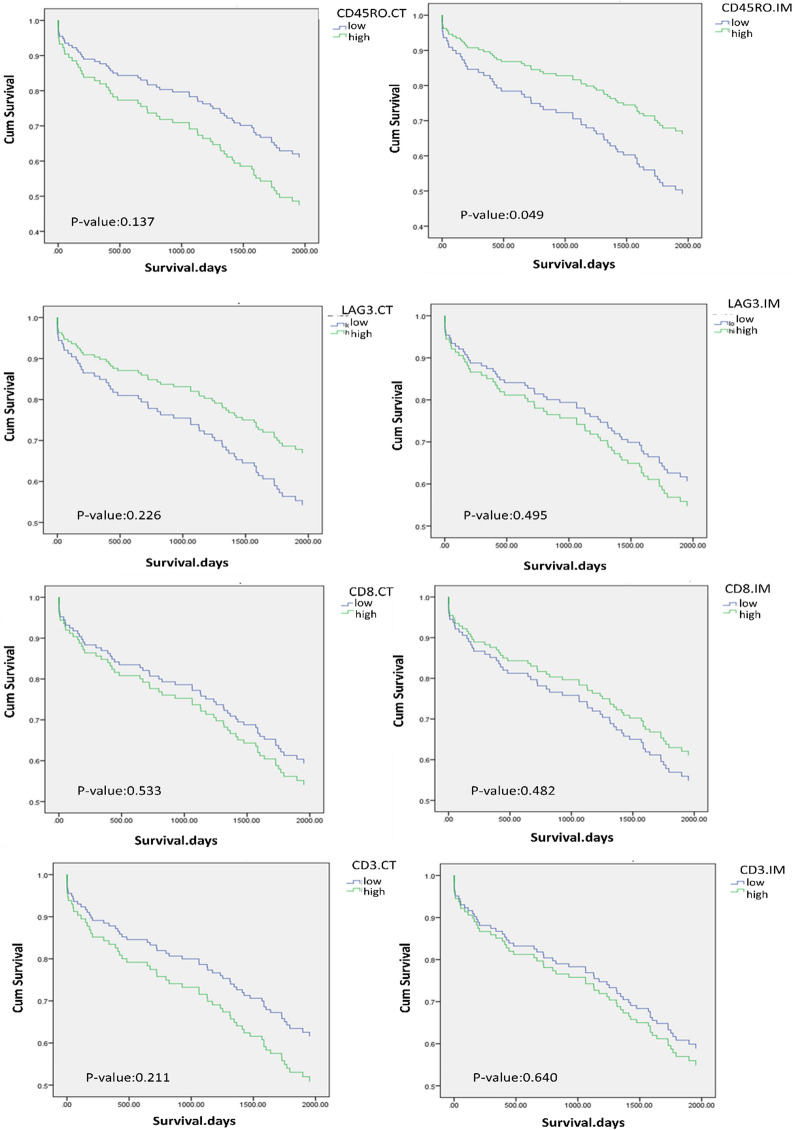
Overall survival (OS) of patients with colorectal cancer based on CD45RO, LAG3, CD8, and CD3 expression tumor tissues. Overall survival (OS) was significantly influenced by CD45RO + TILs. High expression of CD45RO in the IM region of the tumor was significantly associated with better OS (P = 0.049). While there was no significant relationship between CD3, CD8, and LAG3 marker expression and OS of patients (P > 0.05)

To evaluate the effects of CD45RO + , LAG3 + , CD3 + , and CD8 + TILs in CT and IM on OS and DFS in patients with CRC, Cox regression analysis was performed. In univariate model, high score of CD45RO in IM was obtained to be associated with improved OS (hazard ratio [HR] = 0.582, 95% CI = 0.339–0.997, P = 0.049). Whereas no association between LAG3 expression and OS was observed neither in CT nor in IM (Fig. 2). The univariate Cox proportional hazards model for DFS revealed no significant relationship between the expression of investigated markers and the risk of post-operative disease relapse (data not shown). Our analysis also showed that early TNM-stages (HR = 3.529, 95% CI 2.074–6.006, P < 0.001) were associated with improved OS.

All variables in the univariate analysis with a p < 0.1 were then entered into a multivariate Cox regression model. No association was found between the expression of CD45RO, LAG3, CD3, and CD8 and OS, along with the clinicopathological variables (Table 4).

**Table 4:** Univariate Cox regression analysis of overall survival (OS) in patients with colorectal cancer

Parameters	Univariable analysis HR (95% CI)	P-value	Parameters	Univariable analysis HR (95% CI)	P-value
Sex			TLS		
Male	1	0.174	Absent	1	0.415
Female	0.679 (0.388–1.187)		Present	0.760 (0.394–1.469)	
Age			CD45RO.CT		
< 63	1	0.799	Low	1	0.137
≥ 63	1.070 (0.635–1.802)		High	1.511 (0.877–2.603)	
Tumor side			CD45RO.IM		
Right	1	0.119	Low	1	0.049*
Left	1.569 (0.891–2.762)		High	0.653 (0.380–1.123)	
Tumor size			LAG3.CT		
< 5	1	0.549	Low	1	0.226
≥ 5	1.178 (0.689–2.015)		High	0.656 (0.331–1.299)	
Differentiation grade			LAG3.IM		
Low grade	1	0.205	Low	1	0.495
Moderate to high grade	1.399 (0.832–2.353)		High	1.206 (0.704–2.066)	
T stage			CD3.CT		
T1/T2	1	0.060	Low	1	0.211
T3/T4	1.760 (0.976–3.176)		High	1.394 (0.828–2.344)	
Lymph node involvement			CD3.IM		
Absent	1	< 0.001***	Low	1	0.640
Present	2.713 (1.611–4.568)		High	1.133 (0.672–1.908)	
M stage			CD8.CT		
M0	1	< 0.001***	Low	1	0.533
M1	7.443 (3.741–14.808)		High	1.180 (0.702–1.983)	
TNM stage			CD8.IM		
I/II	1	< 0.001***	Low	1	0.482
III/IV	3.529 (2.074–6.006)		High	0.821 (0.473–1.424)	
Lymphovascular invasion (LVI)			CD45RO.IM/CT		
Absent	1	0.663	Low to low	1	0.047*
Present	1.123 (0.666–1.896)		CT high / IM low	1.699(0.773–3.734)	0.187
Perineural invasion			CT low / IM high	0.444(0.209–0.941)	0.034*
Absent	1	0.140	High to high	0.956(0.486–1.880)	0.896
Present	1.576 (0.862–2.881)		LAG3.IM/CT		
Tumor budding			Low to low	1	0.290
Low	1	0.065	CT high / IM low	1.362(0.182–10.175)	0.763
High	1.643 (0.970–2.783)		CT low / IM high	1.509(0.846–2.691)	0.164
			High to high	0.778(0.354–1.710)	0.532

*CT* Center of tumor, *IM* Invasive margin, *TLS* Tertiary lymphoid structure

^*^p < 0.05, ***p < 0.001

### Associations of LAG3 + , CD45RO + , CD3 + , and CD8 + TILs with OS based on primary tumor location

**Fig. 3:**
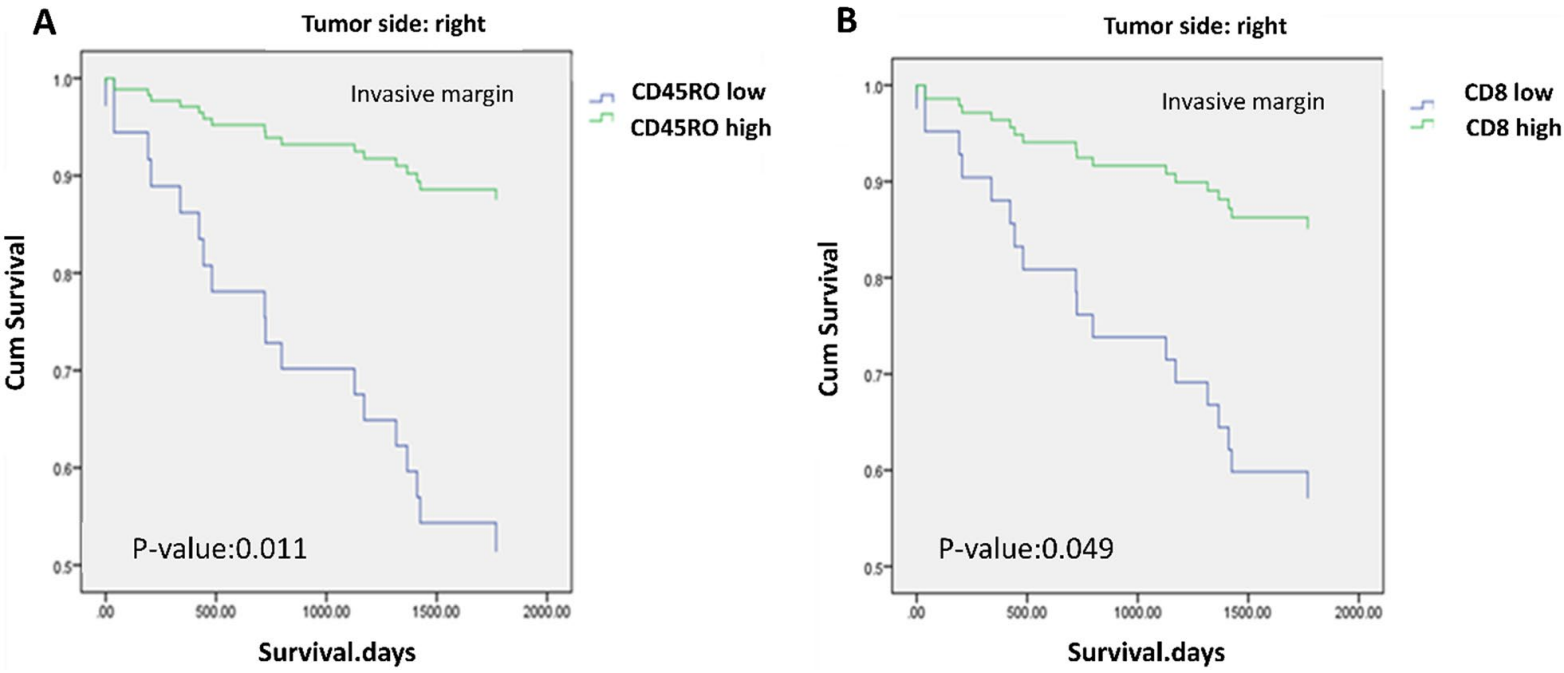
Overall survival (OS) of patients with colorectal cancer based on tumor location. **A** CD45RO expression in IM/ **B** CD8 expression in IM in right-sided tumors. The results obtained from the analysis showed a significant relationship between the high expression of CD45RO in the IM region and better OS in right-sided tumors (P = 0.011). In addition, there was a significant relationship between the high expression of CD8 in the IM region and better OS in right-sided tumors (P = 0.049)

We next analyzed the association between the expression of immune markers and OS based on the tumor location using a univariate Cox regression model. It was observed that there was a significant association between high score of CD45RO in IM and improved OS in right-sided tumors (HR = 0.199, 95% CI 0.057–0.689, P = 0.011). In addition, high score of CD8 in IM was associated with better OS (HR = 0.288, 95% CI 0.083–0.995, P = 0.049), while no correlation was found between CD3 + and LAG3 + TILs and OS (Additional file 1: Table S3 and Fig. 3).

## Discussion

In recent years, the prognostic significance of TILs has been well-documented. Infiltrating TILs, composed of different lymphocyte subtypes, into the TME represents the immune response status between host immune cells and tumors [29]. Many previous studies have shown the association of TILs with tumor development, clinicopathological features of patients, clinical outcomes, and survival in several cancers, including CRC [30]. Of them, Kasurinen et al. investigated the prognostic value of different combinations of CD3 + and CD8 + immune cells in patients with CRC. Their results demonstrated that the infiltration of a large number of CD3 + and CD8 + inflammatory cells in tumor tissue correlated with an improved survival, in many types of cancers [31–33]. In the present study, regarding the importance of memory (CD45RO +) and cytotoxic (CD8 +) lymphocytes in eradication of tumor cells, we attempted to delineate the presence of CD3 + , CD8 + , CD45RO + TILs and the expression of LAG3, as a novel inhibitory marker, in both IM and CT of CRC tissues and then assessed their correlation with clinical outcomes with the focus on tumor location. To the best of our knowledge, none of the previous studies have examined our target markers expressions in CRC and their effects on prognosis based on primary tumor location.

Our results generally showed that CD45RO + lymphocytes, followed by CD3 + lymphocytes, were the most frequent subsets in both areas of CRC tumor tissues, while LAG3 + cells showed the lowest frequency. CD45RO is generally thought to be a marker of memory T cells, which represent effector activity and rapid reaction in TME and can also affect the outcome of the disease [17]. Survival analysis revealed that high score of CD45RO in IM was associated with better OS. Consistently, similar results were reported in several other cancers such as renal cell carcinoma, gastric cancer, breast cancer, and ovarian carcinoma [34–37]. The protective role of CD45RO + T-cells in anti-tumor responses and their association with better prognosis in most cancers might be explained by providing a pool of effector cells or direct killing effect of these cells in TME that repress the proliferation and migration of tumor cells. Additionally, it has been showed that CD45RO + T cells could decrease the production of high-mobility group box 1 (HMGB1) in CRC. Since HMGB1 accelerates cell growth, invasion, and angiogenesis in cancer tissues, CD45RO + lymphocytes might subsequently suppress the proliferation and migration of colorectal tumor cells [38].

In the next step, to investigate the role of primary tumor localization in CRC outcomes, we classified the study population into two groups of right-sided and left-sided tumors. Our survival analysis showed that high CD45RO score in IM in right-sided tumors was directly associated with better survival. Moreover, high score of CD8 in IM was associated with better OS in these patients. These findings further supported previous evidence suggesting that proximal and distal CRC may represent different immunological entities as they also showed differences in epidemiological, pathological, and clinical properties [39].

We also examined the association between the score of CD45RO in CT and IM, with clinicopathological properties in CRC, as well. We observed that low score of CD45RO in CT was correlated with no lymph node metastasis and earlier TNM stages (I/II) in both entire cohort and right-sided tumors. There was also a trend for high score of CD45RO and lower tumor size. Similarly, a study by Hu et al. on patients with lung adenocarcinoma demonstrated that CD45RO + TILs were negatively correlated with tumor size and lymph node involvement [29]. However, controversially, they observed that high density of CD45RO + lymphocytes was associated with earlier TNM-stages.

We also assessed the association of LAG3 score, as a novel ICIs, in both IM and CT of CRC with survival. Overall, although there was a trend for high score of LAG3 in CT and improved OS, we observed no statistically significant association between LAG3 expression and OS. Consistently, most studies, including a meta-analysis on NSCLC, renal cell carcinoma, ovarian cancer, gastric cancer, and a few other cancers, showed that expression of LAG3 on TILs is associated with better survival [40]. The presence of LAG3 + TILs was also associated with longer disease-specific survival in breast cancer patients, as well [41]. On the other hand, some studies reported that LAG3 expression is associated with poor survival. Chen et al., in a study on CRC, demonstrated that patients with high percentage of LAG3 + cells in their tumor tissues had shorter survival compared with those with a low percentage of LAG3 + cells [42]. Considering that LAG3 is an inhibitory immune checkpoint that elicits suppressive immune responses and facilitates tumor escape, such a correlation between the expression of this marker and improved survival may be paradoxical. One hypothesis could be that the low level of LAG3 expression may lead to an exhausted phenotype, but up-regulation of LAG3 may initiate negative feedback of inhibitory signals that creates an active immune environment in the tumor and improve prognosis [43]. However, most previous studies mainly focused on the role of LAG3 in T cell immunity, but a recent study suggested that LAG3 expression correlated with NK-mediated cytotoxicity pathways, and B cell-mediated immunity [44]. Another new aspect of the LAG3 marker is the expression of LAG3 on effector memory T cells with an activated phenotype. In this regard, Slevin et al. showed that LAG3 expression is elevated in the inflamed colonic mucosa of active ulcerative colitis, and stimulated colonic LAG3 + T cells were able to produce IFNγ and IL-17A [45]. This suggests that, at the active inflammation site, LAG3 + cells may have effector rather than suppressor phenotypes. Given this view and the importance of LAG3 expression on memory T cells, we also examine the effect of the simultaneous expression of LAG3 and CD45RO on TILs on survival, although the results did not show any statistically significant relationship (data not shown).

Regarding clinical features, our results showed a relationship between high score of LAG3 in IM with tumor progression (higher T-stage and larger tumor size). We also found that high score of LAG3 in IM was significantly correlated with the absence of TLS formation. Similarly, a previous study on CRC also demonstrated that LAG3 + T lymphocytes in tumor tissue were associated with higher invasion depth and metastasis [42]. A similar result was also reported by Que et al., which showed that LAG3 + T lymphocytes in tumor were associated with larger tumor size in soft tissue sarcoma [46]. Concordantly, considering tumor location, in CT of left-sided tumors, low score of LAG3 was associated with no metastasis and no recurrence. However, there are many studies that could not find a significant association between LAG3 expression and any of the clinical features in NSCLC, CRC, and Hodgkin lymphoma [43, 47, 48]. Controversial to survival analysis, these observations suggested that the LAG3 expression on lymphocytes infiltrated into tumor tissue may trigger suppressive immune responses and facilitates tumor cell proliferation, resulting in disease progression and tumor recurrence or metastasis. This issue should be a topic that warrants further investigation in a larger population.

In conclusion, our results indicated that infiltration of CD45RO + cells in IM is an independent prognostic factor in right-sided colon cancer, but not in left-sided tumors. In addition, higher numbers of LAG3 + TILs in IM correlated with higher T-stage, and low score of LAG3 in CT correlated with the absence of metastasis and recurrence in left-sided colon tumors. These findings implied that the interaction between TILs and tumor cells in TME varies depending on the tumor side. It indicates that tumor location might be an important factor to take into consideration in therapeutic decisions according to the patients' immune status and give physicians a good idea of the prognosis and clinical outcomes of the disease. However, further validation is needed.

## Supplementary Information

**Additional file 1: Table S1. **LAG3 + immune cells in center and invasive margin of colorectal tumors with different clinicopathological features. **Table S2.** CD45RO + immune cells in center and invasive margin of colorectal tumors with different clinicopathological features. **Table S3. **Univariate Cox regression analysis of overall survival (OS) of patients with colorectal cancer based on primary tumor location.

## Data Availability

The data used and/or analyzed during the current study are available in the CRC-ICM data set [49] (https://data.mendeley.com/datasets/h3fhg9zr47).
