# Supplementary material for: Clinicopathological significance and prognostic role of LAG3 + tumor-infiltrating lymphocytes in colorectal cancer; relationship with sidedness
Source: Cancer Cell Int. 2023 Feb 10;23:23. doi: 10.1186/s12935-023-02864-3 (PMC9912542; doi:10.1186/s12935-023-02864-3)
Supplement: Supplementary file 1 — Additional file 1: Table S1. LAG3 + immune cells in center and invasive margin of colorectal tumors with different clinicopathological features. Table S2. CD45RO + immune cells in center and invasive margin of colorectal tumors with different clinicopathological features. Table S3. Univariate Cox regression analysis of overall survival (OS) of patients with colorectal cancer based on primary tumor location. [file 12935_2023_2864_MOESM1_ESM.docx]

**Additional Table 1. LAG3+ immune cells in center and invasive margin of colorectal tumors with different clinicopathological features**

| **Parameters** | **Entire cohort** | | | | | | **Right side** | | | **Left side** | | | **Right side** | | | **Left side** | | |
| --- | --- | --- | --- | --- | --- | --- | --- | --- | --- | --- | --- | --- | --- | --- | --- | --- | --- | --- |
|  | **LAG3- CT** | | **P-value** | **LAG3 - IM** | | **P-value** | **LAG3 - CT** | | **P-value** | **LAG3 - CT** | | **P-value** | **LAG3 - IM** | | **P-value** | **LAG3 - IM** | | **P-value** |
| **Total** | Low | High |  | Low | High |  | Low | High |  | Low | High |  | Low | High |  | Low | High |  |
|  | 104(76.5) | 32(23.5) |  | 55(40.4) | 81(59.6) |  | 45(80.4) | 11(19.6) |  | 56(73.7) | 20(26.3) |  | 22(39.3) | 34(60.7) |  | 32(42.1) | 44(57.9) |  |
| **Sex** | | | | | | | | | | | | | | | | | | |
| Male | 64(61.5) | 19(59.4) | 0.826 | 33(60.0) | 50(61.7) | 0.839 | 26(57.8) | 7(63.6) | 1.000 | 35(62.5) | 12(60.0) | 0.843 | 12(54.5) | 21(61.8) | 0.592 | 20(62.5) | 27(61.4) | 0.92 |
| Female | 40(38.5) | 13(40.6) |  | 22(40.0) | 31(38.3) |  | 19(42.2) | 4(36.4) |  | 21(37.5) | 8(40.0) |  | 10(45.5) | 13(38.2) |  | 12(37.5) | 17(38.6) |  |
| **Age** | | | | | | | | | | | | | | | | | | |
| <63 | 49(47.1) | 16(50.0) | 0.775 | 30(54.5) | 35(43.2) | 0.194 | 20(44.4) | 7(63.6) | 0.253 | 29(51.8) | 9(45.0) | 0.602 | 12(54.5) | 15(44.1) | 0.446 | 18(56.3) | 20(45.5) | 0.353 |
| ≥63 | 55(52.9) | 16(50.0) |  | 25(45.5) | 46(56.8) |  | 25(55.6) | 4(36.4) |  | 27(48.2) | 11(55.0) |  | 10(45.5) | 19(55.9) |  | 14(43.8) | 24(54.5) |  |
| **Tumor side** | | | | | | | | | | | | | | | | | | |
| Right | 45(44.6) | 11(35.5) | 0.371 | 22(40.7) | 34(43.6) | 0.745 |  | | | | | | | | | | | |
| Left | 56(55.4) | 20(64.5) |  | 32(59.3) | 44(56.4) |  |  | | | | | | | | | | | |
| Unknown | 3 | 1 |  | 1 | 3 |  |  | | | | | | | | | | | |
| **Tumor size** | | | | | | | | | | | | | | | | | | |
| <5 | 43(42.2) | 13(40.6) | 0.878 | 28(51.9) | 28(35.0) | 0.052 | 16(36.4) | 3(27.3) | 0.730 | 26(46.4) | 9(45.0) | 0.912 | 10(47.6) | 9(26.5) | 0.109 | 17(53.1) | 18(40.9) | 0.291 |
| ≥5 | 59(57.8) | 19(59.4) |  | 26(48.1) | 52(65.0) |  | 28(63.6) | 8(72.7) |  | 30(53.6) | 11(55.0) |  | 11(52.4) | 25(73.5) |  | 15(46.9) | 26(59.1) |  |
| Unknown | 2 |  |  | 1 | 1 |  | 1 |  |  |  |  |  | 1 |  |  |  |  |  |
| **Differentiation grade** | | | | | | | | | | | | | | | | | | |
| Low grade | 62(59.6) | 14(43.8) | 0.114 | 30(54.5) | 35(43.2) | 0.796 | 27(60.0) | 4(36.4) | 0.190 | 33(58.9) | 10(50.0) | 0.489 | 13(59.1) | 18(52.9) | 0.651 | 17(53.1) | 26(59.1) | 0.604 |
| Moderate to high grade | 42(40.4) | 18(56.3) |  | 25(45.5) | 46(56.8) |  | 18(40.0) | 7(63.6) |  | 23(41.1%) | 10(50.0) |  | 9(40.9) | 16(47.1) |  | 15(46.9) | 18(40.9) |  |
| **T stage** | | | | | | | | | | | | | | | | | | |
| T1/T2 | 33(31.7) | 14(43.8) | 0.211 | 13(23.6) | 34(42.0) | **0.027*** | 16(35.6) | 4(36.4) | 1.000 | 16(28.6) | 10(50.0) | 0.083 | 6(27.3) | 14(41.2) | 0.289 | 6(18.8) | 20(45.5) | **0.015*** |
| T3/T4 | 71(68.3) | 18(56.3) |  | 42(76.4) | 47(58.0) |  | 29(64.4) | 7(63.6) |  | 40(71.4) | 10(50.0) |  | 16(72.7) | 20(58.8) |  | 26(81.3) | 24(54.5) |  |
| **Lymph node involvement** | | | | | | | | | | | | | | | | | | |
| Absent | 66(63.5) | 24(75.0) | 0.228 | 36(65.5) | 54(66.7) | 0.883 | 29(64.4) | 10(90.9) | 0.144 | 35(62.5) | 13(65.0) | 0.842 | 13(59.1) | 26(76.5) | 0.167 | 22(68.8) | 26(59.1) | 0.389 |
| Present | 38(36.5) | 8(25.0) |  | 19(34.5) | 27(33.3) |  | 16(35.6) | 1(9.1) |  | 21(37.5) | 7(35.0) |  | 9(40.9) | 8(23.5) |  | 10(31.3) | 18(40.9) |  |
| **M stage** | | | | | | | | | | | | | | | | | | |
| M0 | 94(90.4) | 30(93.8) | 0.731 | 49(89.1) | 75(92.6) | 0.545 | 41(91.1) | 11(100.0) | 0.575 | 50(89.3) | 18(90.0) | 1.000 | 20(90.9) | 32(94.1) | 0.642 | 28(87.5) | 40(90.9) | 0.714 |
| M1 | 10(9.6) | 2(6.3) |  | 6(10.9) | 6(7.4) |  | 4(8.9) | 0(0.0) |  | 6(10.7) | 2(10.0) |  | 2(9.1) | 2(5.9) |  | 4(12.5) | 4(9.1) |  |
| **TNM stage** | | | | | | | | | | | | | | | | | | |
| I/II | 61(58.7) | 23(71.9) | 0.178 | 32(58.2) | 52(64.2) | 0.479 | 26(57.8) | 10(90.9) | 0.076 | 33(58.9) | 12(60.0) | 0.933 | 11(50.0) | 25(73.5) | 0.073 | 20(62.5) | 25(56.8) | 0.619 |
| III/IV | 43(41.3) | 9(28.1) |  | 23(41.8) | 29(35.8) |  | 19(42.2) | 1(9.1) |  | 23(41.1) | 8(40.0) |  | 11(50.0) | 9(26.5) |  | 12(37.5) | 19(43.2) |  |
| **Lymphovascular invasion** | | | | | | | | | | | | | | | | | | |
| Absent | 61(58.7) | 18(56.3) | 0.81 | 29(52.7) | 50(61.7) | 0.296 | 28(62.2) | 8(72.7) | 0.728 | 33(58.9) | 10(50.0) | 0.489 | 12(54.5) | 24(70.6) | 0.221 | 17(53.1) | 26(59.1) | 0.604 |
| Present | 43(41.3) | 14(43.8) |  | 26(47.3) | 31(38.3) |  | 17(37.8) | 3(27.3) |  | 23(41.1) | 10(50.0) |  | 10(45.5) | 10(29.4) |  | 15(46.9) | 18(40.9) |  |
| **Perineural invasion** | | | | | | | | | | | | | | | | | | |
| Absent | 85(81.7) | 26(81.3) | 0.951 | 43(78.2) | 68(84.0) | 0.394 | 37(82.2) | 11(100.0) | 0.333 | 46(82.1) | 14(70.0) | 0.338 | 18(81.8) | 30(88.2) | 0.698 | 24(75.0) | 36(81.8) | 0.472 |
| Present | 19(18.3) | 6(18.8) |  | 12(21.8) | 13(16.0) |  | 8(17.8) | 0(0.0) |  | 10(17.9) | 6(30.0) |  | 4(18.2) | 4(11.8) |  | 8(25.0) | 8(18.2) |  |
| **Metastasis** | | | | | | | | | | | | | | | | | | |
| Absent | 64(67.4) | 25(89.3) | **0.023*** | 34(68.0) | 55(75.3) | 0.371 | 31(72.1) | 9(90.0) | 0.419 | 30(61.2) | 15(88.2) | **0.039*** | 13(65.0) | 27(81.8) | 0.200 | 20(69.0) | 25(67.6) | 0.904 |
| Present | 31(32.6) | 3(10.7) |  | 16(32.0) | 18(24.7) |  | 12(27.9) | 1(10.0) |  | 19(38.8) | 2(11.8) |  | 7(35.0) | 6(18.2) |  | 9(31.0) | 12(32.4) |  |
| Unknown | 9 | 4 |  | 5 | 8 |  | 2 | 1 |  | 7 | 3 |  | 2 | 1 |  | 3 | 7 |  |
| **Recurrence** | | | | | | | | | | | | | | | | | | |
| Absent | 69(71.9) | 28(93.3) | **0.015*** | 35(72.9) | 62(79.5) | 0.395 | 33(76.7) | 9(90.0) | 0.667 | 34(68.0) | 18(94.7) | **0.027*** | 13(68.4) | 29(85.3) | 0.173 | 21(75.0) | 31(75.6) | 0.954 |
| Present | 27(28.1) | 2(6.7) |  | 13(27.1) | 16(20.5) |  | 10(23.3) | 1(10.0) |  | 16(32.0) | 1(5.3) |  | 6(31.6) | 5(14.7) |  | 7(25.0) | 10(24.4) |  |
| Unknown | 8 | 2 |  | 7 | 3 |  | 2 | 1 |  | 6 | 1 |  | 3 |  |  | 4 | 3 |  |
| **Tumor budding** | | | | | | | | | | | | | | | | | | |
| Low | 71(68.3) | 18(56.3) | 0.211 | 34(61.8) | 55(67.9) | 0.464 | 29(64.4) | 8(72.7) | 0.732 | 39(69.6) | 9(45.0) | **0.05*** | 12(54.5) | 25(73.5) | 0.143 | 21(65.6) | 27(61.4) | 0.704 |
| High | 33(31.7) | 14(43.8) |  | 21(38.2) | 26(32.1) |  | 16(35.6) | 3(27.3) |  | 17(30.4) | 11(55.0) |  | 10(45.5) | 9(26.5) |  | 11(34.4) | 17(38.6) |  |
| **TLS** | | | | | | | | | | | | | | | | | | |
| Absent | 81(77.9) | 23(71.9) | 0.483 | 48(87.3) | 56(69.1) | **0.014*** | 35(77.8) | 7(63.6) | 0.439 | 44(78.6) | 15(75.0) | 0.760 | 19(86.4) | 23(67.6) | 0.114 | 28(87.5) | 31(70.5) | 0.078 |
| Present | 23(22.1) | 9(28.1) |  | 7(12.7) | 25(30.9) |  | 10(22.2) | 4(36.4) |  | 12(21.4) | 5(25.0) |  | 3(13.6) | 11(32.4) |  | 4(12.5) | 13(29.5) |  |
| **Survival** | | | | | | | | | | | | | | | | | | |
| alive | 57(72.2) | 22(27.8) | 0.162 | 34(43.0) | 45(57.0) | 0.468 | 28(73.7) | 10(26.3) | 0.084 | 28(70.0) | 12(30.0) | 0.442 | 15(39.5) | 23(60.5) | 0.967 | 18(45.0) | 22(55.0) | 0.590 |
| dead | 47(82.5) | 10(31.3) |  | 21(36.8) | 36(63.2) |  | 17(94.4) | 1(5.6) |  | 28(77.8) | 8(22.2) |  | 7(38.9) | 11(61.1) |  | 14(38.9) | 22(61.1) |  |

CT: Center of tumor, IM: Invasive margin, TLS: Tertiary lymphoid structure, *p<0.05

**Additional Table 2. CD45RO+ immune cells in center and invasive margin of colorectal tumors with different clinicopathological features**

| Parameters | Entire cohort | | | | | | Right side | | | Left side | | | Right side | | | Left side | | |
| --- | --- | --- | --- | --- | --- | --- | --- | --- | --- | --- | --- | --- | --- | --- | --- | --- | --- | --- |
|  | CD45RO-CT | | P-value | CD45RO-IM | | P-value | CD45RO-CT | | P-value | CD45RO-CT | | P-value | CD45RO-IM | | P-value | CD45RO-IM | | P-value |
|  | Low | High |  | Low | High |  | Low | High |  | Low | High |  | Low | High |  | Low | High |  |
| **Total** | 98(72.1) | 38(27.9) |  | 72(52.9) | 64(47.1) |  | 39(69.6) | 17(30.4) |  | 56(73.7) | 20(26.3) |  | 31(55.4) | 25(44.6) |  | 39(51.3) | 37(48.7) |  |
| **Sex** | | | | | | | | | | | | | | | | | | |
| Male | 57(58.2) | 26(68.4) | 0.271 | 46(63.9) | 37(57.8) | 0.468 | 21(53.8) | 12(70.6) | 0.242 | 34(60.7) | 13(65.0) | 0.735 | 21(67.7) | 12(48.0) | 0.135 | 23(59.0) | 24(64.9) | 0.597 |
| Female | 41(41.8) | 12(31.6) |  | 26(36.1) | 27(42.2) |  | 18(46.2) | 5(29.4) |  | 22(39.3) | 7(35.0) |  | 10(32.3) | 13(52.0) |  | 16(41.0) | 13(35.1) |  |
| **Age** | | | | | | | | | | | | | | | | | | |
| <63 | 49(50.0) | 16(42.1) | 0.408 | 33(45.8) | 32(50.0) | 0.627 | 20(51.3) | 7(41.2) | 0.487 | 29(51.8) | 9(45.0) | 0.602 | 14(45.2) | 13(52.0) | 0.611 | 19(48.7) | 19(51.4) | 0.818 |
| >=63 | 49(50.0) | 22(57.9) |  | 39(54.2) | 32(50.0) |  | 19(48.7) | 10(58.8) |  | 27(48.2) | 11(55.0) |  | 17(54.8) | 12(48.0) |  | 20(51.3) | 18(48.6) |  |
| **Tumor side** | | | | | | | | | | | | | | | | | | |
| Right | 39(41.1) | 17(45.9) | 0.609 | 31(44.3) | 25(40.3) | 0.646 |  |  |  |  |  |  |  |  |  |  |  |  |
| Left | 56(58.9) | 20(54.1) |  | 39(55.7) | 37(59.7) |  |  |  |  |  |  |  |  |  |  |  |  |  |
| Unknown | 3 | 1 |  | 2 | 2 |  |  |  |  |  |  |  |  |  |  |  |  |  |
| **Tumor size** | | | | | | | | | | | | | | | | | | |
| <5 | 43(44.3) | 13(35.1) | 0.335 | 29(40.8) | 27(42.9) | 0.814 | 13(34.2) | 6(35.3) | 0.938 | 28(50.0) | 7(35.0) | 0.248 | 10(33.3) | 9(36.0) | 0.836 | 18(46.2) | 17(45.9) | 0.985 |
| ≥5 | 54(55.7) | 24(64.9) |  | 42(59.2) | 36(57.1) |  | 25(65.8) | 11(64.7) |  | 28(50.0) | 13(65.0) |  | 20(66.7) | 16(64.0) |  | 21(53.8) | 20(54.1) |  |
| Unknown | 1 | 1 |  | 1 | 1 |  | 1 |  |  |  |  |  | 1 |  |  |  |  |  |
| **Differentiation grade** | | | | | | | | | | | | | | | | | | |
| Low grade | 59(60.2) | 17(44.7) | 0.103 | 40(55.6) | 36(56.3) | 0.935 | 23(59.0) | 8(47.1) | 0.41 | 35(62.5) | 8(40.0) | 0.081 | 15(48.4) | 16(64.0) | 0.243 | 24(61.5) | 19(51.4) | 0.37 |
| moderate to high grade | 39(39.8) | 21(55.3) |  | 32(44.4) | 28(43.8) |  | 16(41.0) | 9(52.9) |  | 21(37.5) | 12(60.0) |  | 16(51.6) | 9(36.0) |  | 15(38.5) | 18(48.6) |  |
| **T stage** | | | | | | | | | | | | | | | | | | |
| T1/T2 | 38(38.8) | 9(23.7) | 0.097 | 25(34.7) | 22(34.4) | 0.966 | 16(41.0) | 4(23.5) | 0.209 | 21(37.5) | 5(25.0) | 0.312 | 8(25.8) | 12(48.0) | 0.085 | 16(41.0) | 10(27.0) | 0.199 |
| T3/T4 | 60(61.2) | 29(76.3) |  | 47(65.3) | 42(65.6) |  | 23(59.0) | 13(76.5) |  | 35(62.5) | 15(75.0) |  | 23(74.2) | 13(52.0) |  | 23(59.0) | 27(73.0) |  |
| **lymph node involvement** | | | | | | | | | | | | | | | | | | |
| Absent | 71(72.4) | 19(50.0) | **0.013*** | 43(59.7) | 47(73.4) | 0.092 | 31(79.5) | 8(47.1) | **0.015*** | 38(67.9) | 10(50.0) | 0.155 | 20(64.5) | 19(76.0) | 0.353 | 22(56.4) | 26(70.3) | 0.211 |
| Present | 27(27.6) | 19(50.0) |  | 29(40.3) | 17(26.6) |  | 8(20.5) | 9(52.9) |  | 18(32.1) | 10(50.0) |  | 11(35.5) | 6(24.0) |  | 17(43.6) | 11(29.7) |  |
| **M stage** | | | | | | | | | | | | | | | | | | |
| M0 | 90(91.8) | 34(89.5) | 0.738 | 65(90.3) | 59(92.2) | 0.695 | 36(92.3) | 16(94.1) | 1 | 51(91.1) | 17(85.0) | 0.427 | 28(90.3) | 24(96.0) | 0.62 | 35(89.7) | 33(89.2) | 1 |
| M1 | 8(8.2) | 4(10.5) |  | 7(9.7) | 5(7.8) |  | 3(7.7) | 1(5.9) |  | 5(8.9) | 3(15.0) |  | 3(9.7) | 1(4.0) |  | 4(10.3) | 4(10.8) |  |
| **TNM stage** | | | | | | | | | | | | | | | | | | |
| I/II | 66(67.3) | 18(47.4) | **0.031*** | 40(55.6) | 44(68.8) | 0.114 | 29(74.4) | 7(41.2) | **0.017*** | 35(62.5) | 10(50.0) | 0.329 | 18(58.1) | 18(72.0) | 0.279 | 21(53.8) | 24(64.9) | 0.329 |
| III/IV | 32(32.7) | 20(52.6) |  | 32(44.4) | 20(31.3) |  | 10(25.6) | 10(58.8) |  | 21(37.5) | 10(50.0) |  | 13(41.9) | 7(28.0) |  | 18(46.2) | 13(35.1) |  |
| **Lymphovascular invasion** | | | | | | | | | | | | | | | | | | |
| Absent | 61(62.2) | 18(47.4) | 0.115 | 40(55.6) | 39(60.9) | 0.525 | 26(66.7) | 10(58.8) | 0.573 | 35(62.5) | 8(40.0) | 0.081 | 19(61.3) | 17(68.0) | 0.602 | 21(53.8) | 22(59.5) | 0.622 |
| Present | 37(37.8) | 20(52.6) |  | 32(44.4) | 25(39.1) |  | 13(33.3) | 7(41.2) |  | 21(37.5) | 12(60.0) |  | 12(38.7) | 8(32.0) |  | 18(46.2) | 15(40.5) |  |
| **Perineural invasion** | | | | | | | | | | | | | | | | | | |
| Absent | 81(82.7) | 30(78.9) | 0.617 | 57(79.2) | 54(84.4) | 0.434 | 33(84.6) | 15(88.2) | 1.000 | 45(80.4) | 15(75.0) | 0.750 | 24(77.4) | 24(96.0) | 0.063 | 31(79.5) | 29(78.4) | 0.906 |
| Present | 17(17.3) | 8(21.1) |  | 15(20.8) | 10(15.6) |  | 6(15.4) | 2(11.8) |  | 11(19.6) | 5(25.0) |  | 7(22.6) | 1(4.0) |  | 8(20.5) | 8(21.6) |  |
| **Metastasis** | | | | | | | | | | | | | | | | | | |
| Absent | 67(75.3) | 22(64.7) | 0.241 | 46(68.7) | 43(76.8) | 0.315 | 29(80.6) | 11(64.7) | 0.306 | 35(70.0) | 10(62.5) | 0.575 | 20(66.7) | 20(87.0) | 0.089 | 24(68.6) | 21(67.7) | 0.942 |
| Present | 22(24.7) | 12(35.3) |  | 21(31.3) | 13(23.2) |  | 7(19.4) | 6(35.3) |  | 15(30.0) | 6(37.5) |  | 10(33.3) | 3(13.0) |  | 11(31.4) | 10(32.3) |  |
| Unknown | 9 | 4 |  | 5 | 8 |  | 3 |  |  | 6 | 4 |  | 1 | 2 |  | 4 | 6 |  |
| **Recurrence** | | | | | | | | | | | | | | | | | | |
| Absent | 73(79.3) | 24(70.6) | 0.300 | 53(77.9) | 44(75.9) | 0.782 | 31(83.8) | 11(68.8) | 0.275 | 39(75.0) | 13(76.5) | 1.000 | 22(73.3) | 20(87.0) | 0.313 | 29(80.6) | 23(69.7) | 0.296 |
| Present | 19(20.7) | 10(29.4) |  | 15(22.1) | 14(24.1) |  | 6(16.2) | 5(31.3) |  | 13(25.0) | 4(23.5) |  | 8(26.7) | 3(13.0) |  | 7(19.4) | 10(30.3) |  |
| Unknown | 6 | 4 |  | 4 | 6 |  | 2 | 1 |  | 4 | 3 |  | 1 | 2 |  | 3 | 4 |  |
| **Tumor budding** | | | | | | | | | | | | | | | | | | |
| Low | 62(63.3) | 27(71.1) | 0.392 | 48(66.7) | 41(64.1) | 0.75 | 24(61.5) | 13(76.5) | 0.278 | 35(62.5) | 13(65.0) | 0.842 | 21(67.7) | 16(64.0) | 0.769 | 25(64.1) | 23(62.2) | 0.861 |
| High | 36(36.7) | 11(28.9) |  | 24(33.3) | 23(35.9) |  | 15(38.5) | 4(23.5) |  | 21(37.5) | 7(35.0) |  | 10(32.3) | 9(36.0) |  | 14(35.9) | 14(37.8) |  |
| **TLS** | | | | | | | | | | | | | | | | | | |
| Absent | 78(79.6) | 26(68.4) | 0.168 | 56(77.8) | 48(75.0) | 0.703 | 30(76.9) | 12(70.6) | 0.739 | 45(80.4) | 14(70.0) | 0.361 | 22(71.0) | 20(80.0) | 0.438 | 32(82.1) | 27(73.0) | 0.342 |
| Present | 20(20.4) | 12(31.6) |  | 16(22.2) | 16(25.0) |  | 9(23.1) | 5(29.4) |  | 11(19.6) | 6(30.0) |  | 9(29.0) | 5(20.0) |  | 7(17.9) | 10(27.0) |  |
| **Survival** | | | | | | | | | | | | | | | | | | |
| alive | 61(77.2) | 18(22.8) | 0.115 | 36(45.6) | 43(54.4) | **0.043*** | 27(71.1) | 11(28.9) | 0.739 | 33(82.5) | 7(17.5) | 0.066 | 16(42.1) | 22(57.9) | **0.004**** | 19(47.5) | 21(52.5) | 0.483 |
| dead | 37(64.9) | 20(35.1) |  | 36(63.2) | 21(36.8) |  | 12(66.7) | 6(33.3) |  | 23(63.9) | 13(36.1) |  | 15(83.3) | 3(16.7) |  | 20(55.6) | 16(44.4) |  |

CT: Center of tumor, IM: Invasive margin, TLS: Tertiary lymphoid structure,*p<0.05, **p<0.01

**Additional Table 3. Univariate Cox regression analysis of overall survival (OS) of patients with colorectal cancer based on primary tumor location**

| **Parameters** | **Entire cohort** | | **Right-side** | | **Left-side** | |
| --- | --- | --- | --- | --- | --- | --- |
|  | **Univariate analysis HR (95 % CI)** | **P** | **Univariate analysis HR (95 % CI)** | **P** | **Univariate analysis HR (95 % CI)** | **P** |
| **CD3.CT** | | | | | | |
| Low | 1 | 0.211 | 1 | 0.521 | 1 | 0.359 |
| High | 1.394 (0.828-2.344) |  | 1.356 (0.535-3.441) |  | 1.358 (0.706-2.610) |  |
| **CD3.IM** | | | | | | |
| Low | 1 | 0.640 | 1 | 0.409 | 1 | 0.444 |
| High | 1.133 (0.672-1.908) |  | 0.671 (0.26-1.73) |  | 1.293 (0.670-2.496) |  |
| **CD8.CT** | | | | | | |
| Low | 1 | 0.533 | 1 | 0.938 | 1 | 0.578 |
| High | 1.180 (0.702-1.983) |  | 1.037 (0.409-2.629) |  | 1.205 (0.624-2.327) |  |
| **CD8.IM** | | | | | | |
| Low | 1 | 0.482 | 1 | 0.049* | 1 | 0.499 |
| High | 0.821 (0.473-1.424) |  | 0.288 (0.083-0.995) |  | 1.257 (0.648-2.440) |  |
| **CD45RO.CT** | | | | | | |
| Low | 1 | 0.137 | 1 | 0.761 | 1 | 0.074 |
| High | 1.511 (0.877-2.603) |  | 1.165 (0.437-3.106) |  | 1.860 (0.941-3.674) |  |
| **CD45RO.IM** | | | | | | |
| Low | 1 | 0.049* | 1 | 0.011* | 1 | 0.474 |
| High | 0.582 (0.339-0.997) |  | 0.199 (0.057-0.689) |  | 0.786 (0.407-1.518) |  |
| **LAG3.CT** | | | | | | |
| Low | 1 | 0.226 | 1 | 0.126 | 1 | 0.578 |
| High | 0.656 (0.331-1.299) |  | 0.207 (0.028-1.558) |  | 0.800 (0.365-1.756) |  |
| **LAG3.IM** | | | | | | |
| Low | 1 | 0.495 | 1 | 0.884 | 1 | 0.588 |
| High | 1.206 (0.704-2.066) |  | 0.932 (0361-2.406) |  | 1.204 (0.616-2.353) |  |

CT: Center of tumor, IM: Invasive margin, *p<0.05
